# Supplementary material for: Acceptance and compliance with micronutrient powder (MNP) among children aged 6–23 months in northern Nigeria
Source: PLOS Glob Public Health. 2022 Oct 17;2(10):e0000961. doi: 10.1371/journal.pgph.0000961 (PMC10022258; doi:10.1371/journal.pgph.0000961)
Supplement: S1 Dataset — (ZIP) [file pgph.0000961.s008.zip › S8_Dataset/Spot Checks.zip!/Spot Checks/110715_A_GU_BA_2_SC.pdf]

SPOT CHECK RECORDING FORM

State: __ADAMAWA___ LGA: __GUYUK_______ Community: _GUNDA________

Sex of index child: __FEMALE___ Age of index child (in months): __22 MONTHS_____

Spot Check Information

1. **Date of MNP distribution to household** (circle option below based on LGA name above)**:**
   - **Kebbi:** BirninKebbi&Jega LGA: **June 16**
   - **Kebbi:** Danko-wasagu: **June 17**
   - **Adamawa:**Song, Ganye: **June 18**
   - **Adamawa:**Guyuk **June 19**
2. **Date of Spot Check Observation: ___11/07/2015________________**
3. **Number of sachets remaining**(Ask the caregiver to show you and count the specific number of sachets remaining)**:**

   37

4. **Number of sachets that should remainusing one MNP sachet per day** (Analyst only)**:**

   39

5. **Number of sachets remaining / number of sachets should be remaining**(Analyst only)**:**

__37____ / ___39____ = ___94.9______ %

Probes

Question 1. You have **XX** MNP sachets remaining. Explain how easy or difficult it was to use the MNP product on a daily basis for your child 6 – 23 months? (Circle one answer)

Very Difficult **Difficult** **Easy** **Very Easy**

Question 2. Describe any specific challenges that you faced that made the MNP difficult to use on a daily basis?

I face no challenge in any way using the MNP on daily basis. Though it requires commitment to keep doing it on daily basis

Question 3. Describe specific aspects of the MNP product that you or your child enjoyed that made it easier to use on a daily basis?

Due to the importance of the MNP to improve her health, I don't find it difficult to give her on daily basis. I want her to be healthy and I'm glad to have a product to keep her that way.

Question 4. What questions do you have about the MNP based on your experience using it?

1. Diarrhea, it was a major issue when we started administering the product on her. What do you think might be responsible? Though its virtually absent now but it occurs occasionally.
2. Though I've seen the positive aspect of the MNP such as increased in strength, growing of teeth and increased immunity to diseases or sicknesses. What precisely is the main function of the drug on the child?

Question 5. What specific recommendations do you have that we can do as a program to make the MNP easier to use on a daily basis?

If the product (MNP) can be taken directly without need to be mixed with food. With that, mothers can take it along and give the child at any time of their disposal. That will even help on issues of forgetfulness.
